# Dental caries according to CAST among Zambian adolescents; pattern, socio-demographic and behavioral correlates

**DOI:** 10.1186/s12903-022-02217-y

**Published:** 2022-05-14

**Authors:** Severine N. Anthony, Hawa S. Mbawalla, Febronia K. Kahabuka, Seter Siziya

**Affiliations:** 1 Department of Dental Clinical Sciences, Michael Chilufya Sata School of Medicine, The Copperbelt University, Ndola, Zambia; 2 Department of Orthodontics, Paedodontics and Community Dentistry, School of Dentistry, Muhimbili University of Health and Allied Sciences, Dar es Salaam, Tanzania; 3 Department of Public Health, Michael Chilufya Sata School of Medicine, The Copperbelt University, Ndola, Zambia

**Keywords:** Dental caries, CAST, Socio-demographics, Oral health related behaviors, Adolescents

## Abstract

**Background:**

Dental Caries affect more than half of children and adolescents globally and more so in Africa. Most caries studies in Africa are based on DMFT index which does not assess early carious lesions making early prevention and minimal invasive treatment impossible. This study therefore aimed at determining pattern, socio-demographic and behavioral correlates of dental caries according to Caries Assessment and Treatment Spectrum (CAST).

**Methods:**

Cross-sectional study involving secondary school adolescents in Copperbelt province, Zambia. Socio-demographics and oral health related behaviors were assessed using a structured questionnaire while dental caries was assessed using CAST. Data were summarized as frequency distributions while cross-tabulation with Chi-squire test and adjusted multinomial logistic regression assessed strength and direction of relationship between socio-demographics, oral health behaviors and dental caries. Level of statistical significance was set at 5%.

**Results:**

A total of 1,794 participants were enrolled 54% being females. Total frequency of adolescents with healthy teeth (CAST0-2) was 51.1%, pre-morbidity stages (CAST 3–4) was 24.7%, severe morbidity CAST (6–7) was 6.4% and mortality was 2.7%. The odds of being found with teeth at pre-morbidity stage decreased among male OR (95%CI) = 0.55 (0.44, 0.70) and younger participants OR (95%CI) = 0.77 (0.61, 0.98). Participants in high socio-economic status had lower odds of morbidity OR (95%CI) = 0.69 (0.52, 0.92) while those taking sugary foods five times or more per day had higher odds of morbidity OR (95%CI = 1.52 (1.01, 2.34). The odds of being found at mortality clinical stage of caries was lower among males OR (95% CI) = 0.53 (0.29, 0.96) and those who did not attend to a dentist in the previous year OR (95%CI) = 0.42 (0.23, 0.75), while higher odds OR (95%CI = 2.01 (1.02, 3.97) were among the high socio-economic status.

**Conclusions:**

The proportion of participants with teeth at pre-morbidity and morbidity were high. Socio-demographics and behavioral predictors of dental caries were sex, socio-economic status, frequency of sugary food intake per day and dental visit in the previous year.

## Background

Dental caries remains the most prevalent oral disease globally, despite being a preventable disease [1]. On average, dental caries affect 53.3% of the children globally, 44–54% in Europe, America and Australia and about 59% in Africa and Asia [2]. Wide variations in the prevalence and distribution of dental caries ranging from 8.5 to 88.8% are reported in low to middle -income countries [3, 4]. The variation is explained by diversity in oral health related behaviors, living standards, health systems, and access to preventive health services [4, 5]. The disease is among the leading causes of pain and discomfort and is linked with poor general health, impact on oral health related quality of life, poor school attendance and performance [6, 7].

The main causes of dental caries are oral health related behaviors namely high frequent sugar consumption of 5–7 or more per day and poor oral hygiene [8]. Regular use of fluoridated toothpaste at least twice per day and dental checkup at least once per year significantly reduce occurrence of the disease [9].When these oral health behaviors are acquired during adolescence, they are likely to uphold into adulthood and might have long term consequences on one’s oral health and related quality of life; signifying the importance of assessing and intervening dental caries during this phase of life [10].

Literature demonstrates various socio-demographic differences in the occurrence of dental caries among adolescents, significantly affecting more females than males [4, 11, 12] and older than younger adolescents [3, 4, 13]. While some studies reported significantly higher proportion of adolescents in urban than rural [3] others reported no difference [4, 11]. Some studies report significant impact of socio-economic status on occurrence of dental caries among adolescents [3, 14] but others report no difference [12].

Assessment of dental caries has been predominantly based on Decayed Missing and Filled Teeth (DMFT) index. Recently, improvements in understanding of caries process and change of focus in management of the disease from curative to prevention at early stages and minimal invasive treatment necessitated development of new caries assessment tools. Among the new tools is Caries Assessment and Treatment Spectrum (CAST). CAST meets the current requirements of detecting all stages of the disease including early stages which facilitate proper planning of preventive and curative services [15].

Most of the available published information on dental caries among adolescents in Zambia and Africa at large is based on DMFT index which does not assess early and late stages of caries thus limit proper planning of preventive, surgical and restorative care [16]. This study therefore aims at determining pattern, socio-demographic and behavioral correlates of dental caries at all levels of CAST instrument, among adolescents in Copperbelt Province, Zambia.

## Methods

### Study design, setting and population

A cross sectional study presenting baseline data of the ongoing randomized controlled field trial (RCFT) among 11–19 years old secondary school adolescents in Copperbelt Province, Zambia. Copperbelt province has 2.7 million people ranking second socio-economically after the capital city Lusaka [17]. Its community is heterogenous attracting people from within and outside Zambia, supporting diverse lifestyles and health related behaviors.

### Sample size and sampling technique

A minimum of 1,760 participants for RCFT was calculated assuming; 95% two-sided significant level, 85% power of the study, 5% standard error, 20% expected mean change in caries, 0.001 inter-cluster correlation, cluster size of 80 adolescents and previous mean DMFT of 1.34 [3]. A simple random sampling technique was used to select three out of ten districts of the Copperbelt province. Proportionate sampling guided the number of schools to be selected in each district; 13 out of 21 for Ndola, 5 out of 8 for Masaiti and 4 out of 6 for Mpongwe which were then selected using computer random generated numbers. All adolescents in the school registers during enrollment period (February to May 2021) were assessed for eligibility to participate in this study. Study inclusion criteria were age (10–19 years old), parental/guardian consent and adolescent assent while current fixed orthodontic treatment, use of orthodontic retainers and severe fluorosis were the exclusion criteria. Eligible participants with mixed dentition were not excluded but deciduous teeth were not assessed, and the successor teeth were scored as CAST 9.

### Data collection

Data were collected through self-administered structured questionnaire and clinical examination. The questionnaire was in English made of questions adopted from Global school-based student oral health survey [18] and International Wealth Index (IWI) [19]. It inquired on socio-demographics and oral health related behaviors. The socio-demographic variables were adolescents’ age and sex; parents’ education level and socio-economic status while oral health related behaviors were; frequency of tooth brushing, use of fluoridated toothpaste, intake of sugar-containing drinks and foods in the past 30 days and visiting a dentist in the previous year. Clinical examination for dental caries was done by four trained and calibrated dentists in accordance to CAST field survey protocol [15]. The examiners were subjected to periodic evaluation for their consistency in the clinical assessment of the index throughout data collection. The intra-examiner coefficient of reliability for CAST was in the range 0.80–0.90 while for inter-examiner was 0.80–0.85.

### Data management and analysis

Data entry, cleaning and analysis were done using IBM SPSS for Windows (version 26). The independent variables (demographics of the adolescents, socio-demographics of the parents or guardians, and oral health related-behaviors) were inquired and scored as; age {in years}, sex {1 = male, 2 = female}, and parents’ level of education {1 = No formal education, 2 = Primary education, 3 = Secondary education, 4 = College/University, 5 = I don’t know, 6 = Not applicable}. Adolescents’ age was dichotomized into 11–14 years (early adolescence) and 15–19 years (mid to late adolescence). Parents level of education was dichotomized as {0 = Up to primary and 1 = Secondary and above}. Socio-economic status was inquired using International Wealth Index (IWI) components which included material possession, housing condition; and access to water and electricity utilities. Weights for each item were used to compute total scores and ran Principal Component Analysis (PCA) which was significant {Kaiser- Meyer-Olkin = 0.892; Bartletts’ test significant (*p* < 0.001)}. The adolescents were then categorized into quartiles which in turn was dichotomized as {0 = 1^st^ to 3^rd^ quartile (low to middle SES) and 1 = 4^th^ quartile (high SES)}. Oral health related behaviors (frequency of tooth brushing and use of fluoridated toothpaste) per day were inquired and scored as; {1 = I didn’t, 2 = I did but not every day, 3 = I did once a day, 4 = I did twice a day or more} which was later dichotomized into {0 = twice per day or more and 1 = less than 2 times per day}. Intake of sugar-containing drinks and foods in the past 30 days were inquired and scored as {1 = I didn’t take, 2 = Occasionally per week, 3 = Once per day, 4 = Twice to four times per day, 5 = Five times or more per day} and thereafter dichotomized into {0 = less than 5 times per day and 1 = 5 times or more per day}. Visiting a dentist in the previous year was inquired and scored based on a 3-point scale {1 = I didn’t attend, 2 = I attended once, 3 = I attended twice or more} and thereafter dichotomized into {0 = I didn’t attend and 1 = I attended once or more}.

The dependent variable (dental caries) was scored according to CAST; {0 = Sound, 1 = Sealant, 2 = Restoration, 3 = Caries in enamel, 4 = Caries in dentine (caries-related discoloration in dentine no localized enamel breakdown), 5 = Caries in dentine (distinct cavitation into dentine; the pulp chamber is intact), 6 = Caries in pulp, 7 = Abscess or fistula, 8 = Lost due to caries, 9 = others}. CAST scores were computed to obtain caries maximum score, severity and clinical categories. Maximum CAST scores were obtained as an individual’s highest CAST score for all examined teeth while ignoring CAST 9 and thereafter categorized into five diagnostic thresholds of CAST index namely; {0 = CAST code 0–2 (healthy), 1 = CAST code 3–4 (pre-morbidity), 2 = CAST code 5 (morbidity), 3 = CAST code 6–7 (severe morbidity) and mortality CAST code 8 (mortality). CAST severity score summarized overall caries burden of an individual encompassing CAST codes 3 – 8 and was computed according to Ribeiro et al. (2018) using the formula; CAST severity = 0.25*CAST3 + 1*CAST4 + 2*CAST5 + 4*CAST6 + 5*CAST7 + 6*CAST8 and thereafter classified as follows: {0 = no severity (score = 0), 1 = mild (score 0 < score ≤ 2), 2 = moderate (2 < score ≤ 16), and 3 = severe (score > 16) [20]_._

Socio-demographic, oral health behavior and pattern of dental caries data were summarized and presented as frequency distributions. Cross tabulation with Chi-squire test assessed relationship between dental caries with socio-demographic and oral health behaviors at bivariate level. Adjusted multinomial logistic regression reported as OR (95% CI) were used to assess strength and direction of relationship between dental caries with socio-demographic and oral health behaviors at multivariate level. The level of statistical significance was set at 5%.

Reporting and interpretation of findings in this study conforms with STROBE guidelines [21].

## Results

A total of 2355 secondary school adolescents were invited to participate in this study. Out of these 1909 obtained parents’ written consent to participate. During enrollment period 113 were absent and 2 did not meet inclusion criteria resulting in the final enrollment of 1794 participants. Participants’ socio-demographic characteristics are as shown in Table 1. Females constituted 54% of the sample, 11–14 years old comprised 56% and 54.8% were from urban schools. Concerning parental education attainment, 64.1% of the adolescents’ fathers and 59.7% of their mothers had attained secondary education or higher. More than half of the participants (56.2%) were from high socio-economic status.

**Table 1:** Socio-demographic characteristics of the study participants (n = 1794)

Variable	% (n)
Sex	
Male	46.0 (825)
Female	54.0 (969)
Age	
Early adolescence	56.0 (1004)
Mid to late adolescence	44.0 (790)
Residence	
Rural	45.2 (810)
Urban	54.8 (984)
Father’s education attainment	
Up to primary	35.9 (644)
Secondary and above	64.1 (1150)
Mother’s education attainment	
Up to primary	40.7 (731)
Secondary and above	59.3 (1063)
Socio-economic status	
Low to middle	43.8 (785)
High	56.2 (1009)

Table 2 shows oral health related behaviors of the study participants. Adolescents who reported sugary drinks and foods consumption of five times or more per day comprised 12.2% and 11.7% of the participants, respectively. Use of fluoridated toothpaste of 2 times or more per day was reported by 48.7% and only 26.4% visited a dentist in the previous year.

**Table 2:** Frequency distribution of oral health related behaviors of the study participants

Variable	% (n)
Sugary drinks intake per day in the past 30 days	
Less than 5 times	87.8 (1578)
5 times or more	12.2 (219)
Sugary foods intake per day in the past 30 days	
Less than 5 times	88.3 (1584)
5 times or more	11.7 (210)
Tooth brushing per day	
Less than 2 times	48.6 (872)
2 times or more	51.4 (922)
Use of fluoridated toothpaste per day	
Less than 2 times	51.3 (921)
2 times or more	48.7 (873)
Dental visit in the previous one year	
Attended	26.4 (474)
Did not attend	73.6 (1320)

**Fig. 1:**
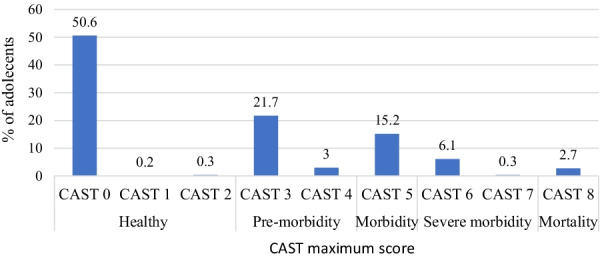
Frequency distribution of study participants’ maximum CAST scores

**Fig. 2:**
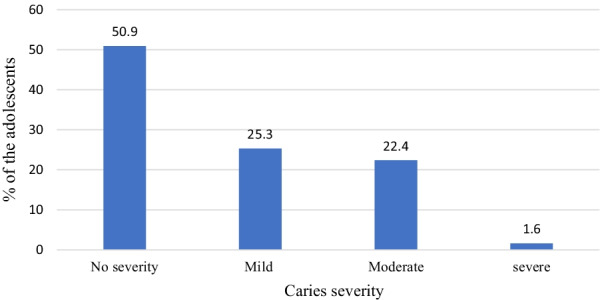
Frequency distribution of study participants’ caries severity

Frequency distribution of the study participants’ maximum CAST scores is as shown in Fig. 1. About half of the adolescents (50.6%) had maximum CAST score of 0, 21.7% CAST 3, 15.2% CAST 5 and 2.7% CAST 8. Total frequency of adolescents with healthy teeth (CAST0-2) was 51.1%, pre-morbidity stages (CAST 3–4) was 24.7%, severe morbidity CAST (6–7) was 6.4% and mortality was 2.7%. Figure 2 shows frequency distribution of study participants’ caries severity. A quarter (25.3%) had mild caries while 1.6% had severe caries.

**Fig. 3:**
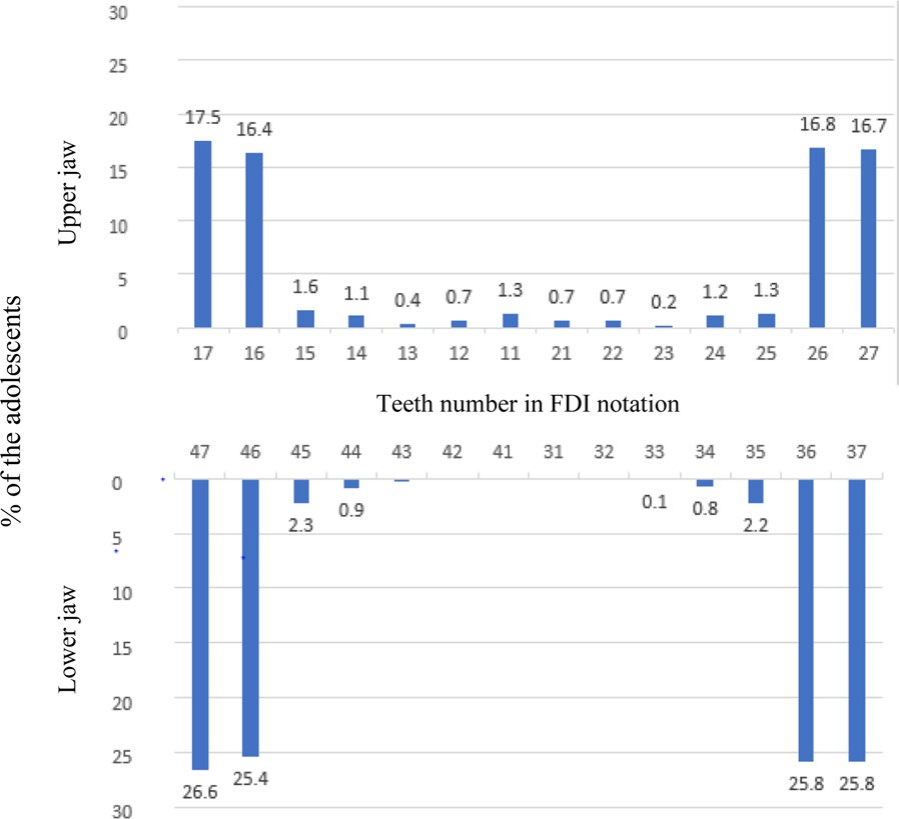
The Pattern of Dental caries by tooth type among adolescents

Figure 3 shows the pattern of dental caries among studied adolescents. The most affected teeth were the first and second molars on both jaws. The lower first molars were more affected (25.4% Right; 25.8% Left) compared to upper first molar (16.4% Right; 16.8 Left). Similar jaw to jaw trend of dental caries pattern was observed for the second molars. However, a reversed trend was observed on anterior segment, where lower anterior teeth were less affected (0.0–0.1%) compared to upper anterior teeth (0.2–0.7%). The most affected tooth in anterior region was upper right central incisor (1.3%). The difference in the proportion of carious teeth between upper and lower molars was statistically significant (*p* < 0.001).

Distribution of the study participants according to CAST clinical categories by socio-demographic variables is as in Table 3. There was statistically significant difference in CAST clinical categories by sex (*p* < 0.001) and socioeconomic status (*p* = 0.007). The proportion of participants with teeth at pre-morbidity and mortality stages were significantly higher among females than males. The proportion of adolescents with teeth at morbidity stage were higher among low to middle than high socioeconomic status while that of pre-morbidity and mortality were higher among high than low to middle socioeconomic status.

**Table 3:** Distribution of the study participants’ CAST clinical categories by socio-demographic variables

Variables	Healthy	CAST clinical categories				*p* value
		Pre-morbidity	Morbidity	Severe morbidity	Mortality	
	% (n)	% (n)	% (n)	% (n)	% (n)	
Sex						
Male	57.1 (471)	19.8 (163)	14.8 (122)	6.3 (52)	2.1 (17)	
Female	46.0 (446)	28.8 (279)	15.5(150)	6.4 (62)	3.3 (32)	< 0.001
Age						
Early adolescence	53.3 (535)	23.1 (232)	15.3 (154)	5.8 (58)	2.5 (25)	
Mid to late adolescence	48.4 (383)	26.6 (210)	14.9 (118)	7.1 (56)	3.0 (24)	0.199
Residence						
Urban	52.6 (518)	23.5 (231)	14.4 (142)	6.6 (65)	2.8 (28)	
Rural	49.3 (399)	26.0 (211)	16.0 (130)	6.0 (49)	2.6 (21)	0.513
Father’s education attainment						
Up to primary	51.1 (329)	24.1 (155)	15.8 (102)	6.7 (43)	2.3 (15)	
Secondary and above	51.1 (588)	25.0 (287)	14.8 (170)	6.2(71)	3.0 (34	0.879
Mother’s education attainment						
Up to primary	51.6 (377)	23.4(171)	17.2 (126)	6.0 (44)	1.8 (13)	
Secondary and above	50.8 (540)	25.5(271)	13.7 (146)	6.6 (70)	3.4 (36)	0.070
Socio-economic status						
Low to middle	50.3 (395)	23.6 (185)	18.0 (141)	6.5 (51)	1.7 (13)	
High	51.7 (522)	25.5 (257)	13.0 (131)	6.2 (63)	3.6 (36)	0.007

Table 4 shows distribution of the study participants’ CAST clinical categories by oral health related behaviors. There was statistically significant difference in CAST clinical categories by frequency of intake of sugary drinks and food (*p* < 0.001) as well as dental visit in the previous year (*p* =  < 0.001). Generally, the proportions of adolescents with healthy teeth were higher among those taking sugary foods or drinks less than 5 times per day while that of morbidity, severe morbidity and mortality were higher among those taking sugary drinks or food 5 times or more per day. The proportion of adolescents with teeth at morbidity stage was higher among those who did not attend to a dentist (16.4%), conversely, mortality was lower (1.8%) in this group compared to those who attended.

**Table 4:** Distribution of the study participants’ CAST clinical categories by oral health related behaviors

Variables	Healthy	CAST clinical categories				*p* value
		Pre-morbidity	Morbidity	Severe Morbidity	Mortality	
	% (n)	% (n)	% (n)	% (n)	% (n)	
Sugary drinks intake/day						
Less than 5 times	52.3 (823)	25.1 (396)	14.3 (226)	5.9 (93)	2.3 (37)	
5 times or more	42.9 (94)	21.0 (46)	21.0 (46)	9.6 (21)	5.5 (12)	< 0.001
Sugary foods intake/day						
Less than 5 times	52.0 (824)	25.6 (406)	14.3 (226)	5.7 (91)	2.3 (37)	
5 times or more	44.3 (93)	17.1 (36)	21.9 (46)	11.0 (23)	5.7 (12)	< 0.001
Tooth brushing/day						
Less than 2 times	51.8 (452)	25.8 (225)	13.4 (117)	6.5 (57)	2.4 (21)	
2 times or more	50.4 (465)	23.5 (217)	16.8 (115)	6.2 (57)	3.0 (28)	0.263
Use of fluoridated toothpaste/day						
Less than 2 times	53.0 (488)	24.8 (228)	13.9 (128)	6.2 (58)	2.1 (19)	
2 times or more	49.1 (429)	24.5 (214)	16.5 (144)	6.4 (56)	3.4 (30)	0.171
Dental visit in the previous year						
Did not attend	49.3 (651)	26.1 (345)	16.4 (217)	6.3 (83)	1.8 (24)	
Attended	56.1 (266)	20.5 (97)	11.6 (55)	6.5 (31)	5.3 (25)	< 0.001

Table 5 show odds ratio and 95% confidence interval of multinomial logistic regression model. Significant factors at bivariate level (sex, socioeconomic status, frequency of sugar drinks, frequency of sugary foods per day and dental visit in the previous one year) and universal confounder (age) were entered in the model. Pearson correlation and deviance were 0.396 and 0.196 respectively indicating that the model fitted well with the data. Likelihood ratio test shows all the variables were significant except frequency of sugary drinks. Male sex decreased likelihood of being found with a carious tooth at pre- morbidity by 46% {OR (95%CI) = 0.55 (0.44, 0.70)} and mortality by 47% {OR (95%CI = 0.53 (0.29, 0.96)}. Young age (11–14) decreased pre -morbidity by 23% {OR (95%CI) = 0.77 (0.61, 0.98), but did not have any effect on other stages of caries. High SES decreased morbidity by 31% {OR (95%CI) = 0.69 (0.52, 0.92)}, however it increased mortality 2.01 times {OR (95%CI = 2.01 (1.02, 3.97)} when compared to low to middle SES. Eating sugary foods 5 times or more increased the likelihood of being found with a tooth at morbidity by 1.52 times {OR (95%CI) = 1.52(1.01, 2.34) and severe morbidity by 1.90 times {OR (95%CI) = 1.90 (1.08, 3.35) but not that of mortality. It also revealed that, those who did not have dental visits were 1.45 times {OR (95% CI) = 1.45 (1.11, 1.91)} more likely to be found with teeth at pre-morbidity stage and 1.57 times {OR (95%CI) = 1.57 (1.12, 2.18)} at morbidity; however, they were 58% less likely {OR (95%CI) = 0.42 (0.23, 0.75)} to be found with a tooth at mortality stage.

**Table 5:** CAST clinical categories parameter estimates at multinomial regression level (reference category – healthy)

CAST clinical categories	Variables	*p* value	OR (95% CI)
Pre-morbidity	Sex (Male)	< 0.001	0.55 (0.44, 0.70)
	Age years (11–14)	0.032	0.77 (0.61, 0.98)
	Socio-economic status (High)	0.267	1.15 (0.90,1.47)
	Frequency of sugar drinks intake (5 time per day)	0.659	1.10 (0.73, 1.65)
	Frequency of sugar foods intake (5 time per day)	0.208	0.76 (0.49, 1.17)
	Frequency of dental visits (did not attended)	0.007	1.45 (1.11, 1.91)
Morbidity	Sex (Male)	0.062	0.77 (0.59, 1.01)
	Age years (11–14)	0.830	1.03 (0.78, 1.37)
	Socio-economic status (High) (High)	0.011	0.69 (0.52, 0.92)
	Frequency of sugar drinks intake (5 time per day)	0.031	1.60 (1.04, 2.46)
	Frequency of sugar foods intake (5 time per day)	0.053	1.52 (1.01, 2.34)
	Frequency of dental visits (did not attend)	0.009	1.57 (1.12, 2.18)
Severe morbidity	Sex (Male)	0.268	0.80 (0.54, 1.19)
	Age years (11–14)	0.136	0.74 (0.49, 1.10)
	Socio-economic status (High)	0.859	0.96 (0.64, 1.46)
	Frequency of sugar drinks intake (5 time per day)	0.164	1.52 (0.84, 2.73)
	Frequency of sugar foods intake (5 time per day)	0.026	1.90 (1.08, 3.35)
	Frequency of dental visits (did not attend)	0.726	1.09 (0.70, 1.68)
Mortality	Sex (Male)	0.038	0.53 (0.29, 0.96)
	Age years (11–14)	0.064	0.57 (0.31, 1.03)
	Socio-economic status (High)	0.043	2.01 (1.02, 3.97)
	Frequency of sugar drinks intake (5 time per day)	0.111	1.90 (0.86, 4.16)
	Frequency of sugar foods intake (5 time per day)	0.070	2.07 (0.94, 4.53)
	Frequency of dental visits (did not attend)	0.003	0.42 (0.23, 0.75)

## Discussion

The current study aimed at assessing the pattern, socio-demographic and behavioral predictors of dental caries among adolescents in Copperbelt province. The strength of this study relies on large sample size and the use of CAST index which assesses all stages of caries process and provides full range of dental caries treatment spectrum for proper planning and management of the disease. Distribution of study participants by socio-demographics was comparable to recent Zambian national demographic and health survey [17] giving a close representation of study population.

The comparatively higher proportion of adolescents with caries reported in this study against previous reported prevalence within Africa is the result of using CAST index instead of traditionally employed DMFT index that exclude early stages of caries in its operational definition of caries. However, totaling advanced stages of caries (CAST 5–7), fissure sealants and restorations (CAST1-2) and extracted teeth due to caries (CAST 8) gives comparable results to previous DMFT studies in Africa [3, 4, 11, 12]. Interestingly, considerable proportion of the participants categorized in pre-morbidity stages (CAST3-4) were found in this study. This category could otherwise not have been captured by the DMFT index and therefore not be addressed in community diagnosis for prevention or comprehensive dental caries management. Furthermore, having large group of adolescents in the category of pre-morbidity caries clinical stage is also a possible indicator of ongoing exposure of risk factors and insufficiency of effective school based oral health promotion activities for this age group in the country. This finding is in line with previous studies which report high prevalence of teeth at pre morbidity stages among adolescents [20], although Velasco et al. 2021 reported the opposite [22]. Untreated obvious decayed teeth were high indicating problems with either seeking or accessing oral health services. Lack of awareness, misconceptions on dental treatment and low capacity of the health systems are the likely main reasons for the observed high prevalence of untreated caries as described in earlier studies [23]. This finding is in line with those of other studies in low to middle income countries [4, 5] but contrary to findings from high-income countries [24].

Generally, the participants of the current study had unfavorable oral health related behaviors where just a handful reported to have visited a dentist in the past year and most likely for therapeutic and not dental check-up. Lack of established community based oral health prevention and promotion programs, dental services, inadequate dental supplies and materials and lack of enough qualified oral health care staff may explain the observed low dental attendance. Likewise, a substantial proportion of these adolescents had insufficient fluoride exposure as reported by more than half of them not using fluoridated toothpaste at least twice daily and therefore not likely to benefit the preventive action of toothbrushing and use of fluoridated toothpaste [25]. Affordability to oral hygiene products and fluoridated toothpaste in most low-middle income countries could give a plausible explanation to the low usage. Our results are in line with other findings in developing countries, which report low proportion of the adolescents practicing recommended frequency of dental checkup, toothbrushing and use of fluoridated toothpaste [13, 26]. Although relatively low proportion reported to consume sugary foods and drinks five times or more per day, when this behavior is coupled with insufficient oral health promotion behaviors mentioned above, their risk to caries is definite. Our results on sugary foods and drinks intake are comparable to previous studies in low to middle income countries [27] but lower than those reported in high-income countries [23].

Concerning sociodemographic predictors of dental caries, we found sex of the adolescents and their family socio-economic status being the only statistically significant factors associated with caries in this group of adolescents, indicating oral health disparity in this community. The fact that higher percent of females had one or more teeth at pre-morbidity and mortality, indicate increased and ongoing risk exposure of this subgroup. The differences by sex could be attributed to differences in eating behaviors, saliva flow rate, and genetics in males and early eruption of permanent teeth in females [28]. The current findings agree with previous studies among adolescents which reported lower odds of caries prevalence among males than female [12, 23]. Likewise, the high socio-economic status participants being less likely to be at morbidity stage and at the same time more likely at mortality (lost teeth due to caries) than their comparative group is an indicator of social gradient in health where the advantage group enjoy the existing best oral health care available in the community. Middle and higher socioeconomic status participants who are expected to afford dental care, end up accessing tooth extraction services mainly and therefore reducing morbidity but increasing mortality. The unexpected increase in mortality among those who can afford dental care could be explained by late treatment seeking behavior, lack of emphasis on restorative treatment in most low to middle income countries. Similar findings are reported by other researchers [7, 13].

Regardless of the social state; the odds of being found with teeth at advanced caries stages namely morbidity, severe morbidity and mortality was higher among participants reporting sugary food intake of 5 times or more. The observation is supported by a well-established evidence-based role of high frequency of intake of cariogenic substrate in causation of dental caries [29]. The finding is in agreement with those of other studies which report a positive relationship between frequency of intake of sugary foods and dental caries [29].

As reported earlier that the well to do participants had most of their teeth in mortality clinical caries state; it is not a surprising that the odds of being found with caries at pre-morbidity and morbidity stages of caries increased among those who did not attend to dentist. Oral health services provided in most developing countries including Zambia are inclined towards exodontia explaining the irrationally increased risks for being found with a tooth at mortality stage among those who attended to a dentist mindless of the socio-economic group.

### Study limitations

Interpretation of the findings of this study needs to be done within the limitation of a cross sectional study and the reduced accuracy of diagnosis of early carious lesions in the field environment.

## Conclusion and suggestions

The proportions of participants with teeth at pre-morbidity and morbidity were high implying potential increase in teeth mortality if effective preventive measures are not put in place. First and second molars were the most affected teeth on both jaws. Sex, socioeconomic status, frequency of intake of sugary food and drinks per day and visit to a dentist in the previous one year predicted both occurrence and severity of dental caries.

High proportion of pre-morbidity and morbidity calls for follow up to prevent the lesions from developing to frank cavitation. Control of intake of sugar diet is the key intervention in prevention of dental caries in this population due to strong positive relationship between caries and sugar diet intake. Concerted efforts among all oral health stake holders need to put in place to minimize unnecessary tooth extraction among patients attending for dental care.

## Data Availability

Data generated and analyzed are available from the authors (Corresponding author) upon reasonable request and with permission of Zambia Health Research Authority and Muhimbili University of Health and Allied Science, Tanzania.

## Abbreviations

CAST: Caries assessment and treatment spectrum
DMFT: Decayed filled missing teeth
FDI: Federation dental international
IRB: Institutional review board
IWI: International Wealth Index
MUHAS: Muhimbili university of health and allied sciences
PCA: Principal component analysis
RCFT: Randomized controlled field trial
SES: Socio-economic status
WHO: World health organization
